# Degradation Monitoring of HDPE Material in CO_2_-Saturated NaCl Environment through Electrochemical Impedance Spectroscopy Technique

**DOI:** 10.3390/ma14112823

**Published:** 2021-05-25

**Authors:** Hafiz Usman Khalid, Mokhtar Che Ismail, Norlin Nosbi

**Affiliations:** Department of Mechanical Engineering, Universiti Teknologi PETRONAS, Perak 32610, Malaysia; mokhtis@utp.edu.my (M.C.I.); norlin.nosbi@utp.edu.my (N.N.)

**Keywords:** HDPE, CO_2_ exposure, EIS, dielectric, degradation monitoring

## Abstract

Extensive damage due to saturated seawater and CO_2_ exposure under high temperature and pressure in high-density polyethylene (HDPE) has been studied by Infrared Spectroscopy (FTIR), Differential Scanning Calorimetry (DSC), Thermogravimetric Analysis (TGA), Field Emission Scanning Electron Microscope (FESEM), and Electrochemical Impedance Spectroscopy (EIS). The degradation of square-shaped HDPE samples having 1 mm thickness was investigated at 70 bars with 60, 75, and 90 °C separately for three weeks in an autoclave chamber. A clear indication of aging was observed in terms of chain scission by the formation of the methyl group (1262 cm^−1^), and the appearance of degradation products, including the alcohol and hydroxyl groups. The decline in glass transition temperature (T_g_), melting point (T_m_), and crystallinity (*X_c_*) result from branching and formation of degradation products in the aged samples. TGA results reveal that the degradation shifts the characteristic temperatures (T_5%_ and T_10%_) to lower values compared to virgin HDPE. FESEM images show clear surface cracks and rough patches after 3 weeks. The *X_c_* value increased due to chain mobility at higher temperatures (90 °C). The impedance is relatively high 10^11^ ohms.cm^−2^ for a virgin sample, but it drops down to 10^9^ and 10^6^ after degradation. Impedance and dielectric loss were correlated, and the significance of dielectric loss was observed at lower frequencies. These characterizations will contribute to more efficient and detailed evaluation criteria for degradation monitoring.

## 1. Introduction

Pipelines are used for the transportation of crude oil and natural gas in the oil and gas industries. In upstream production, pipeline integrity is affected by corrosion due to contaminants in the process fluid such as CO_2_, H_2_S, and microbes. CO_2_ corrosion (sweet corrosion) and H_2_S (sour corrosion) are the most prevalent forms of corrosion, influenced by many factors such as CO_2_ and H_2_S content, water chemistry, temperature, flow velocity, and material surface condition [1,2].

Typical industrial practices employ cost-effective mitigation methods of material selection, corrosion inhibition and chemical treatments, metallic and non-metallic linings, and other appropriate options [3,4]. Recently, a potential option of non-metallic pipe (NMP) for combating internal pipeline corrosion is assessed. The use of non-metallic pipes (NMP) in oil and gas production and transportation is deemed to be promising based on the improved material properties that are accepted as viable and reliable solutions to reduce corrosion [5]. However, as a part of monitoring and maintenance management, continuous monitoring of the installation is required.

NMP liners provide a potential corrosion management solution in cases where the long-term reliability of chemical corrosion inhibition systems is not suitable. Sometimes, the inhibitor consumption rate is so high that it becomes more expensive than a liner over the pipeline’s lifetime [2,6]. NMP has been implemented in pipelines mainly due to its corrosion resistance to the process environment [7,8,9]. However, these materials degrade as a result of their interaction with the process environment [10,11]. The most common polymer material used in the pipeline industry includes high-density polyethylene (HDPE), polyamide (PA11, PA12), and polyvinylidene fluoride (PVDF) [8,12,13]. 

Thermoplastic-lined pipes have been proved to be commercially viable in one of the reports given by Atkins Boreas [13]. Thermoplastic liners (TPL) have been used effectively to control downhole failures in oil and gas production compared to other mitigation strategies, including coatings and chemical treatment. The usage of TPL was cost-effective, as proved by the case studies in Canada, Basin, and Bahrain [8]. 

The degradation or aging susceptibility of thermoplastics depends on the environmental factors and polymer chemical structure (molecular weight, type of bonds, crystallinity) [14,15]. Thermoplastics have an inherent property to allow gases, vapors, and liquids to pass through them. The environmental and temperature factors radically influence them [16,17]. In general, degradation of polymers occurs due to permeation, absorption, and oxidation, particularly in the immersed condition of the internal pipeline [9,18]. However, the degradation is a time-dependent and slow process.

Aging studies are intended to accelerate the degradation chemistry, and the resulting degradation stage is often monitored with supporting chemical analysis such as IR signatures, discoloration, crosslink state, melting behavior, crystallinity, and morphological characterization [19,20,21,22]. It is well known that the degradation proceeds through chain scission, degree of branching, and crosslinking, as evident by the decrease in the molecular weight [15]. 

Permeation is the prominent aspect of the degradation of pipelines due to the expo-sure of acidic gases and immersion in hydrocarbons, including acids in the presence of temperature and pressure [23,24,25,26,27]. In the presence of hydrocarbons, polyolefins swell as they both have a similar chemical structure. Polyamide degrades due to the presence of water at higher temperatures [28].

**Figure 1:**
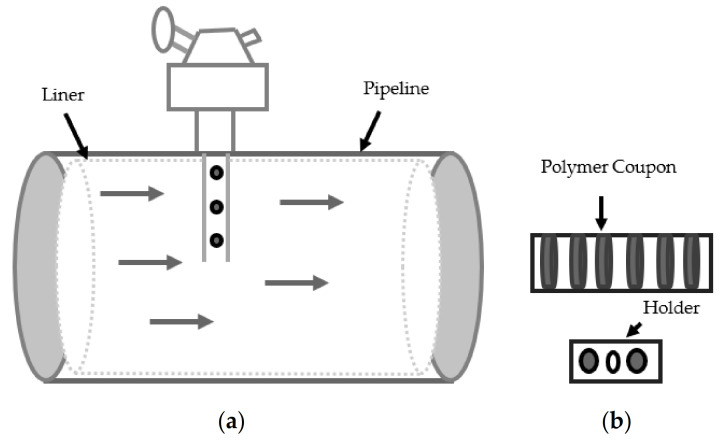
(**a**) Polymer coupon in the pipeline, (**b**) coupon and holder.

Permeation under high temperature-pressure working conditions containing hydrocarbons and acidic gases limits the NMP’s operational envelopes. Thus, condition-based monitoring through systematic evaluation of degradation phases is critical for successfully using NMP materials. Furthermore, permeation is a slow process and requires sensitive monitoring methods. Currently, test coupons are used for monitoring NMP degradation as shown in Figure 1. These coupons are inserted in pipelines and then retrieved and evaluated using molecular weight change as a measuring indicator [25,29,30]. The coupon method provides a qualitative measurement of the degradation mechanism and is incapable of monitoring the progression of damage [31,32]. 

The EIS technique is proposed in this research article to evaluate HDPE performance after the degradation in terms of impedance property. It can be used for corrosion rate measurements, corrosivity monitoring, coating integrity measurements, and reaction mechanism investigations based on impedance property. Due to immersion in the HCl and chloride solution, coatings degrade, which is illustrated by the formation of a capacitive loop in the Nyquist diagram, and the electrolyte has penetrated through the coating and reached the metal surface [33,34]. Corrosion pitting was seen in the embedded steel reinforcement due to the presence of different chloride concentrations [35]. Similar studies have been carried out to study the corrosion and aging behavior of X65 steel, KOH electrolyte, and hard metals [36,37,38].

For the life prediction of the NMP, a quantitative correlation of the damage phases is indispensable. There is a research gap in on-field evaluation, such as post-installation (in-service) degradation modes for NMP due to permeation. Our objective is to establish a correlation between degradation chemistry with the HDPE polymer’s critical physical and dielectric properties. It can be useful for quantitative measures to identify the degradation stages in the material after permeation damage. Hence, there is a need to provide an evaluation criterion that can contribute to a more systematic and detailed assessment process.

## 2. Materials and Methods

### 2.1. Sample Preparation

The commercial HDPE polymeric sheet of 1 mm thickness has been provided by ARJ Development Sdn Bhd, Perak, Malaysia. It has been cut into square samples with dimensions of 23 mm. CO_2_ gas cylinders were provided by AGS Sdn Bhd, Penang, Malaysia while NaCl was taken from the Materials lab of CCR in UTP, Perak, Malaysia. Five samples were used as a reference for different characterizations, and the remaining ones were used for degradation purposes. The HDPE sheet and CO_2_ gas which were used in the experiment with the following properties are shown below in Table 1 and Table 2.

### 2.2. Degradation/Aging of HDPE

The aging of the HDPE was carried out in the HPHT (high-pressure, high temperature) autoclave containing the 3.5% NaCl solution pressurized with CO_2_ gas. The parameters are shown in Table 3.

The experiment was designed so that the virgin samples were immersed in CO_2_-saturated 3.5 wt% NaCl solution in the high-temperature, high-pressure autoclave. The evaluation of polymeric properties includes chemical structure, crystallinity, thermal stability, microstructure, and dielectric property using FTIR, DSC, TGA, FESEM, and EIS techniques. 

### 2.3. Characterization Techniques

#### 2.3.1. Fourier Transform Infrared Spectroscopy (FTIR)

FTIR was performed using a PerkinElmer frontier model spectrometer installed in the Universiti Teknologi Petronas, Seri Iskandar, Malaysia, equipped with attenuated total reflectance (ATR) at the scanning range of 500–4000 cm^−1^ frequency and resolution of 4 cm^−1^. Based on the spectral recordings, the variations that occurred were analyzed: formation/disappearance, increase/decrease, and displacement of various bands. The virgin samples of HDPE usually contain five spectral bands [39,40].

#### 2.3.2. Differential Scanning Calorimetry (DSC)

DSC is a thermoanalytical technique and examines heat effects associated with phase transitions and chemical reactions as a function of temperature. Thermal analysis was carried out to measure the glass transition temperature (T_g_), melting temperature (T_m_), melting enthalpy, and % crystallinity (*X*_c_). Around 10.08 mg sample mass was taken for the analysis. The sample was heated twice from −80 to 170 °C with a heating and cooling rate of 10 °C/min rate in a nitrogen gas atmosphere. The thermal route consists of two cycles. The first cycle eliminates the thermal history and residual solvents, while the second one is for getting sharp T_g_. The T_g_ is calculated using the TA analysis software, and it is in sub-ambient temperatures [41]. 

Equation (1) was used to carry out the *X_c_* (% crystallinity) measurement for the reference sample:

(1) $$X_{c}=\frac{\Delta H_{m}(cal)}{\Delta H_{s}}\times 100$$

where $\Delta H_{m}(cal)$ is the calculated heat (J/g) of fusion values and $\Delta H_{m}(s)$ is the standard heat of fusion values for 100% crystalline HDPE. The given value for crystalline HDPE in the literature is 293 J/g [42,43,44]. 

#### 2.3.3. Thermogravimetric Analysis (TGA)

The thermal stability of HDPE samples was studied using TGA analysis. PerkinElmer STA 6000 simultaneous thermal analyzer installed in the Universiti Teknologi Petronas, Seri Iskandar, Malaysia, was employed at a temperature range of 450–500 °C with 10 °C/min heating rate under nitrogen atmosphere.

#### 2.3.4. Field Emission Scanning Electron Microscopy (FESEM)

A Zeiss SUPRA 55 VP microscope installed in the Universiti Teknologi Petronas, Seri Iskandar, Malaysia, was employed to investigate the morphological properties of virgin and aged polymers after being coated with gold using a sputter coater (Emitech K550X). 

#### 2.3.5. Electrochemical Impedance Spectroscopy (EIS)

A potentiostat/galvanostatic Autolab model PGSTAT30 was used for the EIS measurements. It is employed by the 3-electrode arrangement cell with 3.5 wt% NaCl solution. Stainless steel, Ag/AgCl, and the coated polymer were used as a counter, reference, and working electrode, respectively. The measurement frequencies range from 10 MHz to 100 kHz with 10 mV amplitude. 

**Figure 2:**
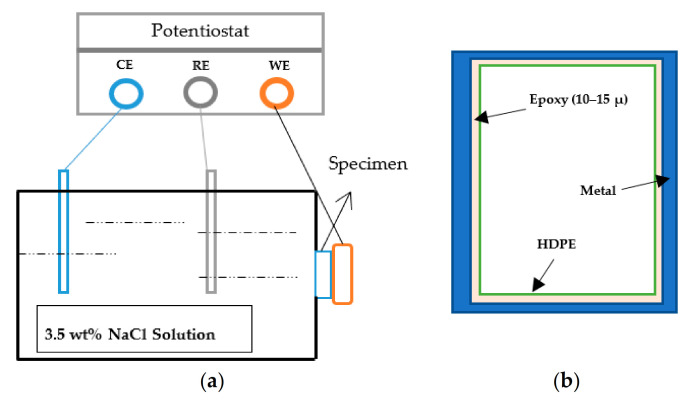
(**a**) Assembly of EIS setup, (**b**) prepared sample.

There are two main ways to plot impedance spectra, Bode and Nyquist. Nyquist is sensitive to changes; imaginary impedance was plotted versus the real part of impedance, while for Bode, the total impedance is plotted versus frequency and the phase shift. For the EIS test, we use our samples as a coating. Therefore, four carbon steel plates have the exact dimensions as the HDPE samples that were used for this purpose. The epoxy adhesive was applied on the sides of the HDPE sample and metal plate making a 10–15 microns thick layer. The prepared sample for the EIS test and the EIS setup is shown in Figure 2a,b. 

## 3. Results and Discussion 

### 3.1. Structural Modifications and Chemical Composition 

**Figure 3:**
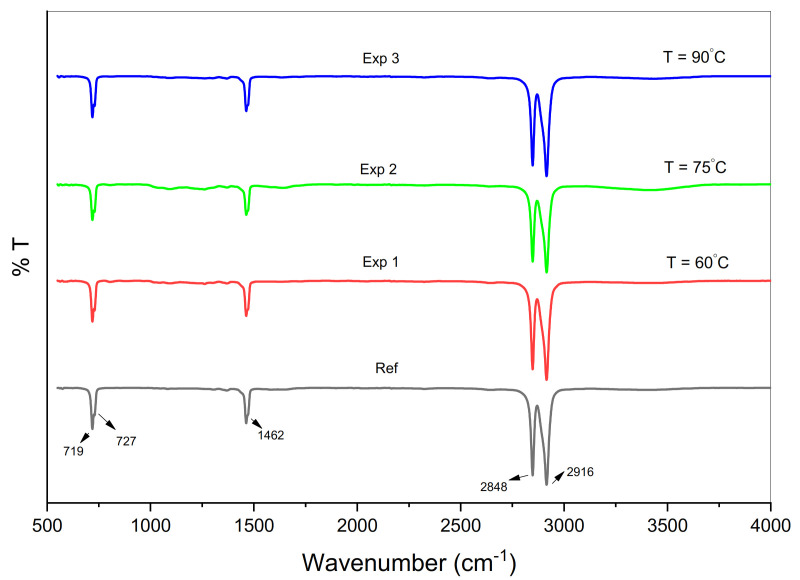
FTIR spectra for virgin and aged samples.

**Figure 4:**
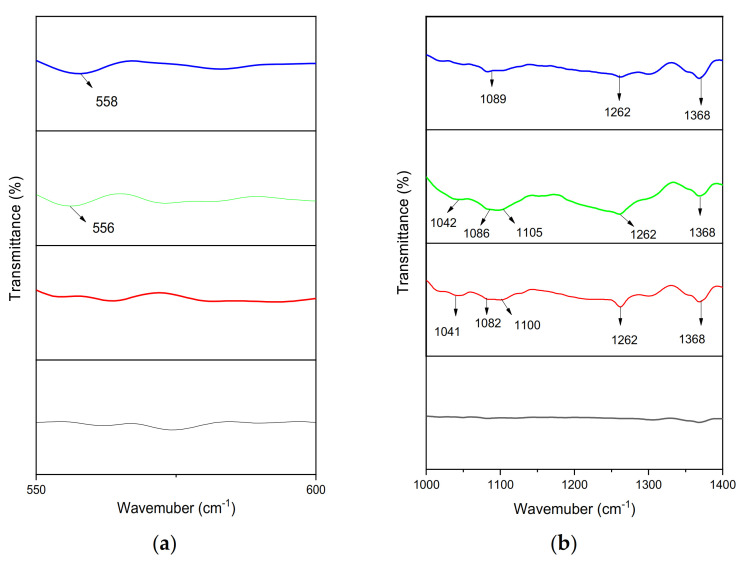
FTIR spectra (**a**) 550–600, (**b**) 1000–1400.

**Figure 5:**
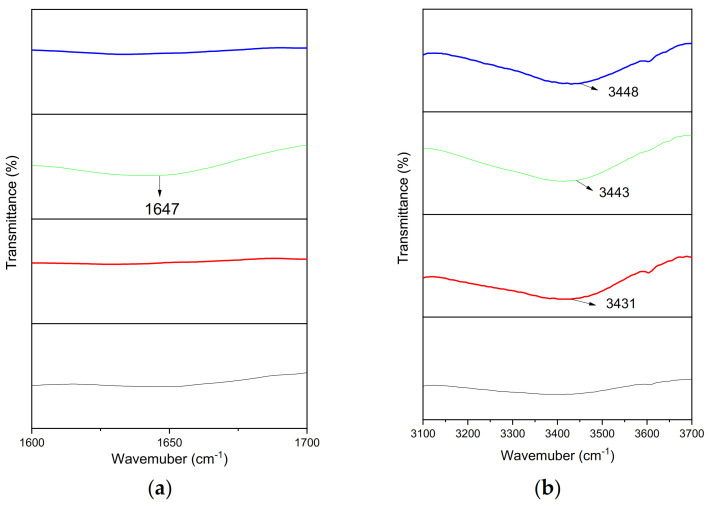
FTIR spectra (**a**) 1600–1700, (**b**) 3100–3700.

The evaluation of chemical and structural changes was based on the comparison of the pre-and post-exposure spectra. The peaks around 2915 and 2848 cm^−1^ show the asymmetric and symmetric vibration of CH_2_ [45], CH_2_ (methylene) bending observed at 1462 cm^−1^. The virgin sample of HDPE always contains a strong peak at 1462 cm^−1^ because it is associated with methylene and CH_2_ scissoring. Vibration in the amorphous part and deformation in the crystalline phase was observed at 718 cm^−1^ and 729 cm^−1^ [19,46]. Figure 3 represents five different peaks in the spectra of 1 mm-thick HDPE material. The difference in absorbance bands occurred after the exposure experiments in the autoclave chamber, shown in Figure 4a,b, and Figure 5a,b. 

As expected, the extent of the observed variations is stronger at higher temperatures. The sample degradation occurred largely through chain scissions in the soft amorphous phase and was usually reflected through an increase in the chain mobility of this region. There are four regions 550–600, 1000–1400, 1600–1700, and 3100–3700 cm^−1^ in which polymer aging was shown in terms of different absorption bands as shown in Figure 4a,b, and Figure 5a,b. The appearance of different functional groups and their assigned spectral bands are shown in Table 4. 

#### 3.1.1. Chain Scission

The bands around and 720 and 1468 are due to the vibrational deformation band of the methylene group. The peaks around 725–730 demonstrate connected methylene; as the exposure temperature increases, the wavenumber shifted from lower to higher values (729 cm^−1^), showing the decline of molecular chain length [47,48]. The chain-breaking de-fines the aging of HDPE with the characteristic group including 1368 cm^−1^ [49]. The peak around 1368 cm^−1^ is due to external hydrogen vibrations of CH_2_ [19,34] or wagging deformation of methylene groups. The appearance of peaks in these bands can be attributed to the presence of side-chain branches of PE [45,50]. The band around 1262 cm^−1^ (methyl) shows the process of chain scission because it appears in all the experiments since methyl served as the end group in molecular chains [46,47].

#### 3.1.2. Degradation Products

The formation of degradation products after the high temperature and pressure exposure has been shown in different absorption bands. The appearance of bands around 1041 and 1042 shows the C-O stretching in primary alcohol or O-H deformation in alcohol [51]. The absorption bands appear around 1082, 1086, and 1089, respectively, are associated with C-O stretching in alcohol, and this is due to the presence of saturated seawater under high temperature in the surroundings [52]. The bands around 1100 and 1105 are due to C-O vibration within the alcohol as a degradation product [53,54]. The appearance of the bands around 556 cm^−1^ and 558 cm^−1^ are due to the contact of the process medium with the polymer surface as the solution contains chlorides, and it is also evident by the rough surface patches and cracks in FESEM images [55]. The band around 1647 cm^−1^ in experiment 2 shows the formation of unsaturation groups in the polymer and demonstrates that O-H bending is due to water absorption [42]. The peaks that appeared between 3100 and 3700 are associated with the presence of liquid water within the sample or hydroxyl group and intramolecular hydrogen bonding [19,20]. As the temperature increases within the experiments, the hydroxyl group shows increased thermal degradation [19,20,44,56]. As the temperature increases from 60 to 75 °C, the water is absorbed into the polymer surface with cracks, as shown by the bands. The hydroxyl group shows the acidic functional group due to acidic gas (CO_2_) [57].

The change in the peak values of almost all functional groups supports the conformational change on the polymer surface. The pressure and the NaCl solution degrade the material by leaving the characteristic signature of oxygen, chlorine, and hydroxyl; this also causes breakage of tie molecules followed by the chain layering and crystalline content as well as the formation of microcracks [42]. 

### 3.2. Thermal and Crystallinity Analysis

**Figure 6:**
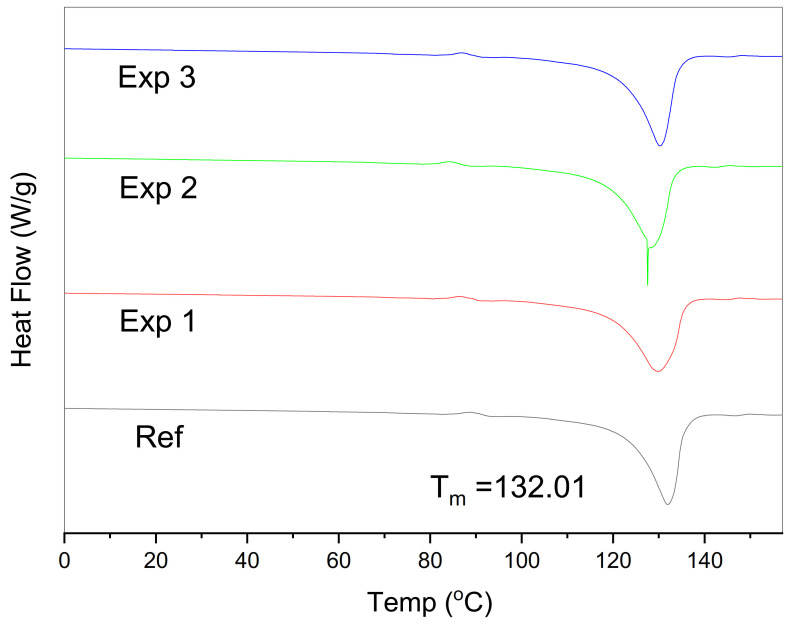
DSC heating curves for virgin and aged samples.

**Figure A1:**
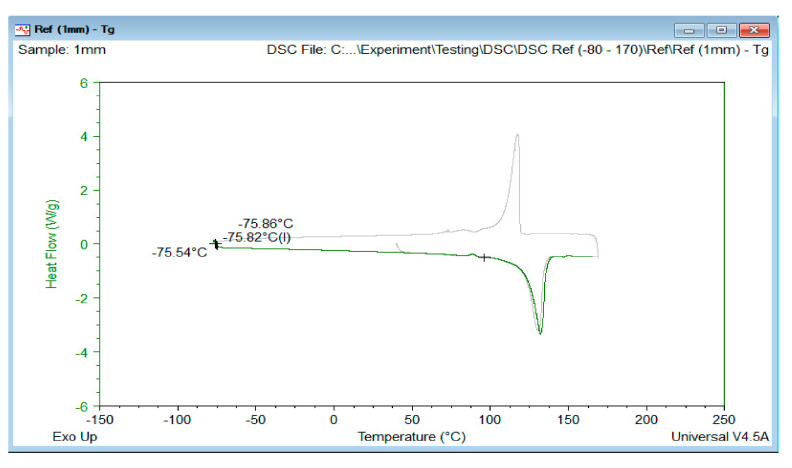
T_g_ of virgin HDPE.

**Figure A2:**
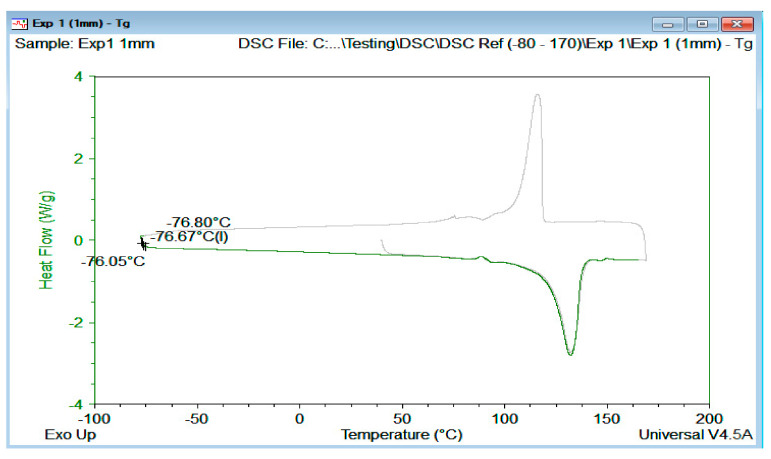
T_g_ of HDPE after Experiment 1.

**Figure A3:**
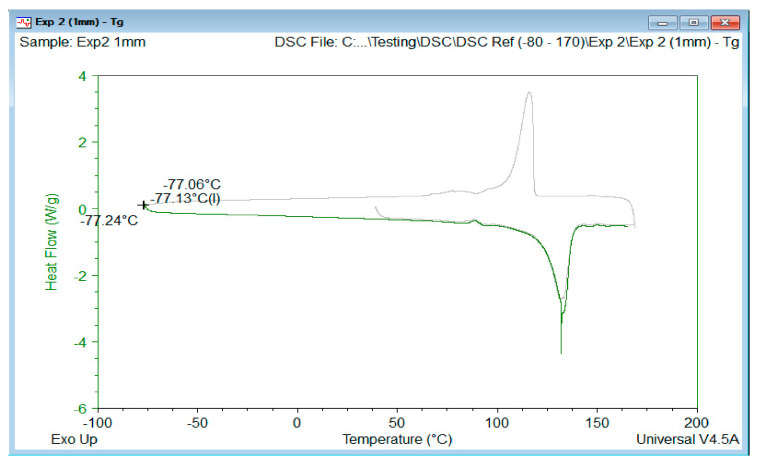
T_g_ of HDPE after Experiment 2.

**Figure A4:**
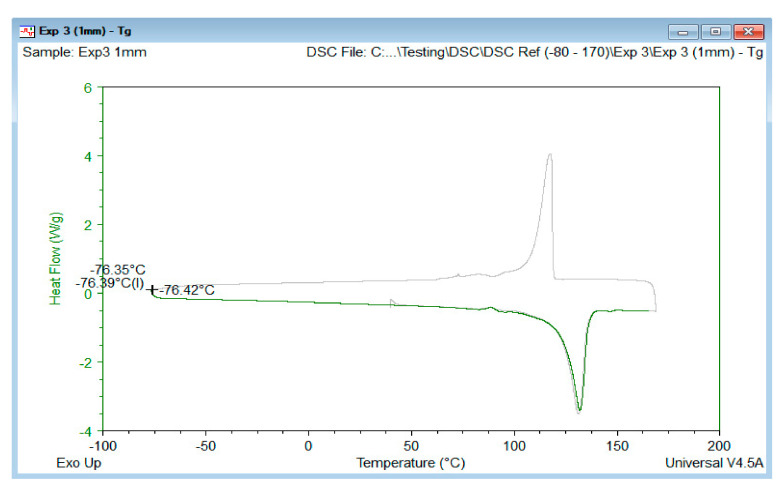
T_g_ of HDPE after Experiment 3.

The T_m_ of the virgin sample is 132.01 °C. The step between 80 and 100 °C is typically due to smaller crystals, and probably they were made due to some processing. It can be due to the inclusion of additives, pigments, or simple thermal or physical processing [58] of the material before being tested in the DSC, as shown in Figure 6. Figure A1, Figure A2, Figure A3 and Figure A4 have been shown in Appendix A for representing T_g_ values. The experimental values for T_m_, T_g,_ and *X_c_* were in Table 5. 

The decrease in crystallinity is mainly due to the formation of degradation products (alcohol, hydroxyl) and branching [59,60,61]. Branched polyethylenes have lower crystallinity and melting temperature as compared to linear ones [62]. Furthermore, chain scission increases the HDPE chain mobility, which increases the *X_c_* at high temperatures [59]. The increase in crystallinity was observed at high temperatures (90 °C) and is attributed to the enhanced chain mobility in the amorphous phase, which favors morphological rearrangements [63,64]. 

The absorption of CO_2_ in HDPE is greater in experiments 1 and 2 [23]. The small amount of decrease in T_g_ is due to CO_2_ absorption at 60 and 75 °C, and it is evident by the reduction in crystallinity. Due to induced aging, the crystallinity values become higher, and it restricts the diffusion of CO_2_, also evident by the slight increase in T_g_ [65]. The increase in CO_2_ diffusion followed by the rise in temperature as pressure is constant throughout the experiments [66]. T_m_ and T_g_ both show the decreasing trend due to branching that occurred in the HDPE samples after the degradation. 

The neat HDPE crystallizes at 119 °C, and in our case, it is 117 °C [52]. Branching within the polymer relates to density and further with the crystallinity of the polymer. After thermal treatment and CO_2_ exposure, chain scission occurred, and the sample lost its crystallinity which can also be seen by the decrease in T_m_. Chain mobility increases at higher temperatures which tends to enhance the crystallinity values [51]. 

More side chain branches decrease the crystallinity as well as the melting temperature. The crystallinity of the polymer directly relates to the density; an increase in density induces higher crystallinity values [67]. DSC results are affected by the branching, which lowers the T_5%_ and T_10%_. The formation of new degradation products plays the role of defect centers, disturbing the reorganization and chain folding during the crystallization process [59,61]. This fact produces imperfect crystallites as reflected by *X_c_*, T_5%,_ and T_10%_ values.

### 3.3. Thermal Stability Analysis

**Figure 7:**
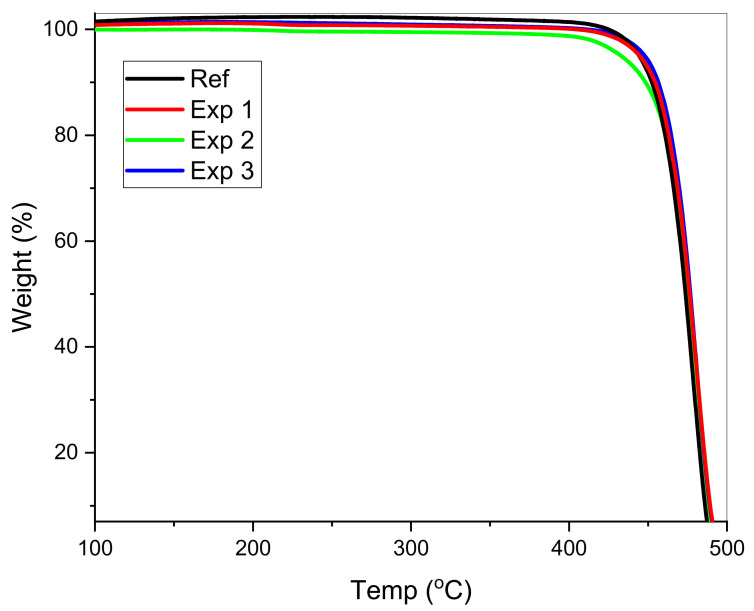
Thermograms for virgin and aged samples.

Representative TGA curves for virgin and aged samples after the CO_2_ exposure at 60, 75, and 90 °C have been shown in Figure 7. We will use T_5%_ and T_10%_ (temperature for 5% and 10% weight loss) to demonstrate the aging within the samples. The respective values for weight loss against temperature are shown in Table 6. All the curves exhibit only one degradation step attributed to the chain scission mechanism of polyolefin material thermal degradation [59]. HDPE dissociation at lower temperatures follows C-C bond breakage, while C-H dissociation occurred at high temperatures [53]. 

The weight loss in terms of T_5%_ and T_10%_ is more significant in experiments 1 and 2; this is due to the chain breaking and scission in the aged sample supported by the DSC thermograms. The values for T_5%_ and T_10%_ decreased in the first two experiments due to a drop in crystallinity factor [44]. Alcohol and hydroxyl groups appeared as a degradation product, as seen by the FTIR spectrum. As soon the branching disappears at a high temperature, the temperature drops down, as shown in Table 6 and Figure 7 [59,68]. The material got thermally stable due to higher crystallinity values at higher temperatures in experiment 3. The changes in the thermal decomposition temperatures show the clear effect of polymer structure (branch content) on a thermogravimetric curve. 

### 3.4. Morphological Properties

**Figure 8:**
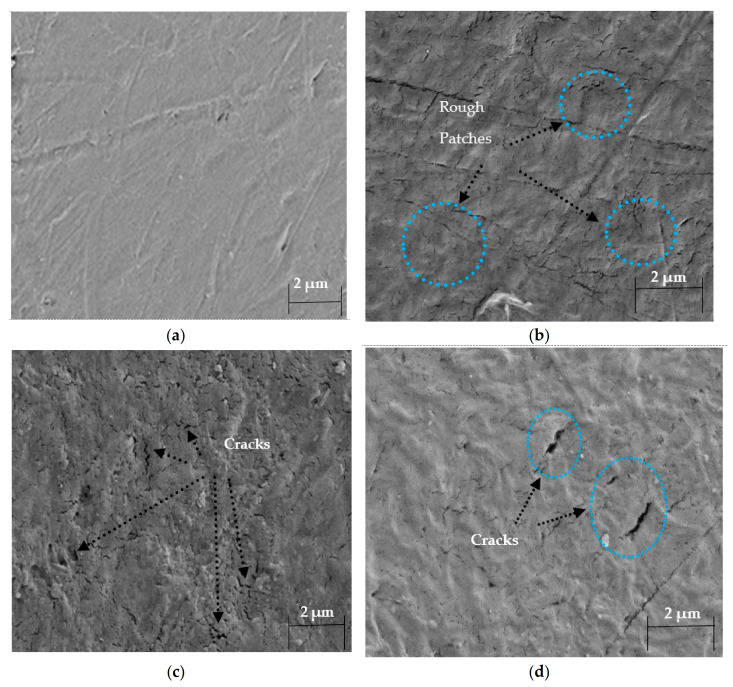
FESEM micrographs for virgin and aged samples.(**a**) Reference, (**b**) Experiment 1, (**c**) Experiment 2, (**d**) Experiment 3.

Figure 8a–d shows the micrograph taken from the virgin sample and aged samples after three weeks of aging in the autoclave chamber.

The material loses its strength due to aging in the aggressive environment with an acidic gas (CO_2_) under high temperature and pressure. Microcracks and surface damage were identified due to thermal degradation and the presence of chlorides in the autoclave chamber [44]. The appearance of roughness in experiment 1, while surface cracks in experiments 2 and 3 illustrate the attack of NaCl solution and CO_2_ in the autoclave chamber under high temperature and pressure [69,70]. 

### 3.5. Impedance Analysis 

**Figure 9:**
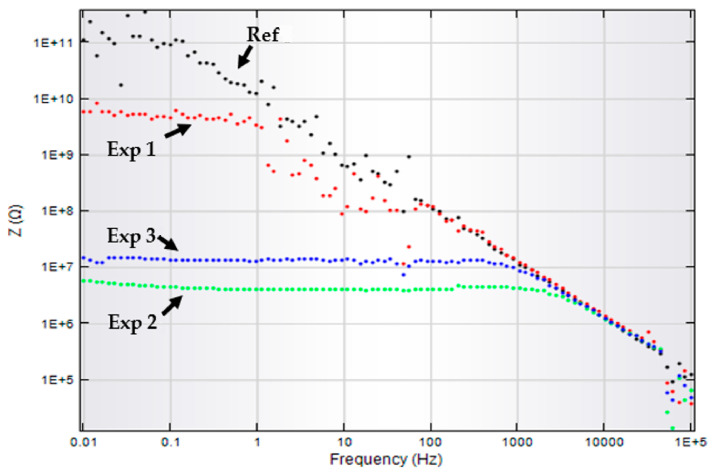
Bode plot for virgin and aged samples.

EIS investigation was carried out to study the effect of CO_2_ exposure and simulated seawater solution on virgin and aged HDPE samples. Figure 9 shows the corresponding Bode plot for virgin and aged samples.

**Figure 10:**
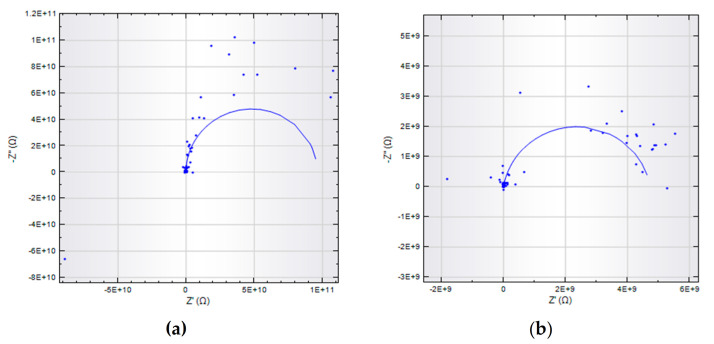

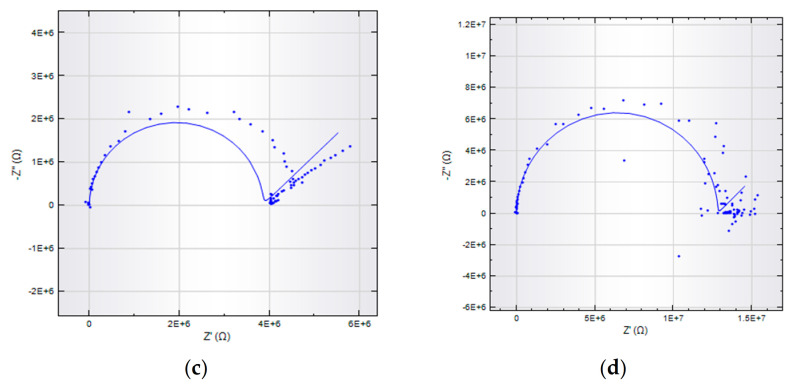
Modeled Nyquist plots for virgin and aged samples (**a**) Reference, (**b**) Experiment 1, (**c**) Experiment 2, (**d**) Experiment 3.

**Figure 11:**
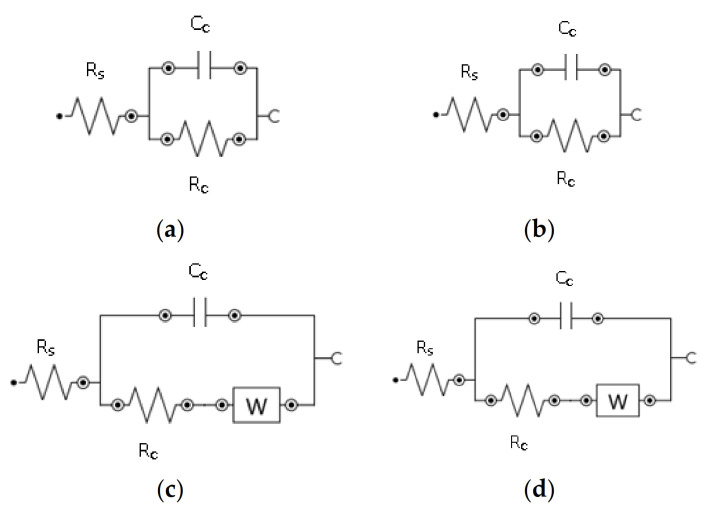
EEC of virgin and aged samples. (**a**) Reference (**b**) Experiment 1 (**c**) Experiment 2 (**d**) Experiment 3.

The impedance decreased in experiments 1 and 2 due to the chain scission, which occurred due to CO_2_ exposure and saturated seawater in the autoclave, as shown in Figure 9. The aged material loses its strength as compared to the virgin sample. Initially, the virgin sample indicates very high resistance (10^11^) at low frequency. The impedance values higher than 10^7^ illustrate excellent surface protection of the sample [33]. The half semicircle at high frequency was ascribed to coating protection performance. The coating impedance decreases when the material degrades in the autoclave, and the capacitance loop can be seen in the Nyquist plot. Coating impedance starts decreasing from 10^11^ to 10^9^ then 10^6^ ohms.cm^−2^ for samples aged at 60 and 75 °C temperatures. Coating resistance (R_c_) decreases while the capacitance increased, as shown in the Nyquist plot. However, the resistance gets higher at 90 °C, and this behavior also shows us the loss in capacitance. Z_w_ represents the Warburg impedance, which resulted from the diffusion of ions from the electrolyte to the electrode interface. The modeling of EIS data was carried out using the NOVA software, an equivalent electrical circuits (EEC) were used to analyze the degradation of coatings quantitatively. Nyquist plots and the EEC circuits have been shown in Figure 10 and Figure 11, while their respective circuit element values are shown in Table 7.

After CO_2_ exposure, the crystallinity decreases, indicating aging within the sample as well as the occurrence of roughness and microcracks. Nyquist plots show us the diffusion mechanism within the sample at lower frequencies as illustrated by the Warburg diffusion mechanism. The data points are dispersed as seen in the Nyquist plot and this electrochemical noise is probably due to high sample thickness. Thick and high-quality coatings have almost infinite resistance with low capacitance [71,72]. Due to this feature, we get minimal current values, and resistive elements in the model dominate [72]. The difference in impedance values is quite prominent in lower frequencies. We have seen the diffusion mechanism in experiments 2 and 3, while the Z_w_ increases at higher temperatures. The EEC values for virgin and aged samples are shown in Table 7. 

#### Dielectric Properties 

The real and imaginary parts of impedance have been gathered from the Nyquist plot. Our aim here is to relate the impedance with the dielectric property of the HDPE material. HDPE has an ordered structure and has less free volume for polarizability. We can calculate the dielectric loss from Equations (2–4) and hope to see what changes before and after the CO_2_ exposure experiment within the HDPE material. 

We will calculate the real and imaginary permittivity from complex impedance, which in turn gives us dielectric loss [73]. Table 8 is showing the dielectric factor at different low frequencies.

(2) $$\cdot C=\frac{\varepsilon_{o}\varepsilon_{r}A}{t},$$

(3) $$\cdot \varepsilon^{\prime }=\frac{t}{\omega A\varepsilon_{o}}\times \frac{Z^{\prime \prime }}{{\left(Z^{\prime }\right)}^{2}+{\left(Z^{\prime \prime }\right)}^{2}},$$

(4) $$\varepsilon^{\prime \prime }=\frac{t}{\omega A\varepsilon_{o}}\times \frac{Z^{\prime }}{{\left(Z^{\prime }\right)}^{2}+{\left(Z^{\prime \prime }\right)}^{2}.}\;,$$

where $\varepsilon_{o}$ (vacuum permittivity) = 8.85 × 10^−12^, $\varepsilon_{r}$(material‘s permittivity) = 2.4, 

Area (A) = 0.023 m^2^, thickness (t) = 0.001 m, $\varepsilon^{\prime }$ (real permittivity), $\varepsilon^{\prime \prime }$ (imaginary permittivity or dielectric loss), tanδ (loss tangent or dissipation factor), $Z^{\prime }$ (real impedance), $Z^{\prime \prime }$ (imaginary impedance).

The decrease in dielectric loss factor with gradually increasing frequency is the common characteristic of dielectric material. The analysis with temperature shows that the dielectric loss of HDPE increases in the low-frequency region until 75 °C; after that, it decreases at much higher temperatures [74,75]. In dielectric materials, the dielectric losses occur due to the dipolar molecules. Dielectric loss increases followed by the rise in aging temperature.

The relation between impedance and dielectric loss is contrary. Higher values of dielectric loss have been seen in experiments 1 and 2 to explain the aging of HDPE after the CO_2_ exposure and thermal treatment. The hydroxyl group (-OH) in non-polar polymers increases the dielectric loss factor and is associated with the size and dipole orientation for small molecules [76]. The mobility of segments in the amorphous phase and within the small portions of macromolecules are affected by polymer crystallization. The values of dielectric loss increase as the polymer crystallization occurs. This is mainly due to the transition of polar portions of macromolecules into crystallites [77]. 

## 4. Conclusions

Our investigation aimed to monitor the influence of temperature and CO_2_ exposure on the impedance property and dielectric loss of our HDPE sample. HDPE samples were subjected to aging in the autoclave chamber at 60, 75, and 90 °C for three weeks. With aging in the presence of saturated seawater and acidic gas (CO_2_) in the chamber, HDPE aged by showing the following symptoms in terms of color, crystallinity, chemical structure, morphology, impedance, and dielectric loss. Thermal aging leads to slow and irreversible changes in terms of material properties.

The appearance of a methyl group, hydroxyl, and alcohol explains the molecular chain scission in HDPE at 1262, 3400–3500, and 1462 cm^−1^. Water absorption into the polymer matrix around 1647 cm^−1^ also explains the polymer aging within the chamber after the exposure. The magnitude of dielectric loss rises by increasing the concentration of the polar molecule, which is proved by the presence of the O-H absorption band and hydroxyl group in our FTIR spectra around 1647 cm^−1^ and within 3400-3500. 

DSC thermograms validate the decrease in % crystallinity with a change in T_g_. The reduction in crystallinity occurred due to the breakage of chains inside the polymer structure at 60 and 75 °C. Moreover, it was enhanced at 90 °C due to higher chain mobility which helps in molecular rearrangements. CO_2_ absorption is favored at temperatures 60 and 75 °C, which is why there is a slight increase in T_g_ here. Thermoplastics such as HDPE show less CO_2_ absorption due to their semi-crystalline feature. New peaks have confirmed the extent of physical aging in the FTIR spectrum and FESEM images. Surface cracks and roughness were observed in the FESEM images. At 75 °C, the C-X, (X-halide) band appears, which indicates the roughness on the surface. 

The impedance of the virgin sample decreased after the intensive exposure of CO_2_ and simulated seawater in the autoclave. The presence of a diffusion tail in the Nyquist diagram indicates that the material aged. The dielectric loss property is related to interfacial polarization. These effects are caused by the boundaries between the amorphous and crystalline regions. The impedance and dielectric properties differ indirectly in relation to one another, and as crystallinity increases, the dielectric loss decreases. Using these two crucial properties, we can easily demonstrate the aging mechanism within the HDPE material, and it will facilitate us to monitor and assess the HDPE material degradation in oil and gas pipelines. 

The outcomes of the dielectric property contribute to a more inclusive and systematic post-permeation monitoring approach. In this way, we can establish some acceptance requirements for the remaining polymer life. A comprehensive assessment is provided, showing the challenges allied with the monitoring of the polymer liner material in the pipeline as it relates to the lifetime prediction requirement. This can provide a lifeline for the assets in the oil and gas industries. 

## Figures and Tables

**Table 1:** Physical properties of CO_2_.

Material	Density (g/cm^3^)	Melting Temperature (°C)	Glass Transition (°C)	Dielectric Constant	Dielectric Strength (MV/m)
HDPE	0.95	125–130	−78	2.3–2.4	18.9–160

**Table 2:** Properties of HDPE material.

Gas	MW (g/mol)	Density (kg/m^3^)	Critical Temperature (°C)	Crystal Structure	Purity (%)
CO_2_	44.01	1.977	31.1	Trigonal	99.9

**Table 3:** Experimental Design Parameters.

Material	Parameters		Characterization
HDPE	-	-	FTIR Evaluation of the chemical changes within the structure
	Pressure	70 bars	DSC Evaluation of crystallinity and melting temperature
	Temperature	60, 75, 90 °C	TGA Evaluation of thermal stability and mass loss
	Duration	21 days	FESEM Evaluation of morphological properties
	-	-	EIS Evaluation of impedance and dielectric properties

**Table 4:** Spectral bands for virgin and aged samples.

Wavenumber (cm^−1^)				Assigned Vibrational Bands/Functional Groups
Reference	Exp 1	Exp 2	Exp 3	
		556	558	C-Cl bending vibration [47]
719	719	719	719	CH_2_ rocking vibration (amorphous phase) [19,48]
727	728	729	729	CH_2_ rocking vibration (crystalline phase) [49,50]
	1041	1042		C-O stretching (alcohol)/O-H deformation in alcohol [51]
	1082	1086	1089	C-O stretching (alcohol) [52]
	1100	1105		C-O stretching vibration (alcohol) [53,54]
	1262	1262	1262	CH_3_ vibration [52,53]
	1368	1368	1368	CH_2_ wagging (amorphous) [55]
1463	1463	1463	1463	CH_2_ scissoring (amorphous phase) 45,46]
		1647		O-H bending (water absorption) [48]
2848	2848	2848	2848	CH_2_ symmetric stretching [51]
2916	2916	2916	2916	Asymmetric stretching of CH_2_ [51]
	3431	3443	3448	Hydroxyl group [19,20]

**Table 5:** Thermal properties for virgin and aged samples.

Sample	Melting Temp (T_m_)	Melting Enthalpy	Glass Transition (T_g_)	Crystallinity (%*X_c_*) ^a^
Ref	132.01	177.6	−75.82	60.6
Exp 1	130.24	173.8	−76.67	59.3
Exp 2	129.01	168.6	−77.13	57.5
Exp 3	129.85	170.9	−76.39	58.3

^a^ Observed heat of fusion divided by 293 J/g.

**Table 6:** Thermal degradation temperatures for virgin and aged samples.

Characteristic Temperatures	Ref	Exp 1	Exp 2	Exp 3
T_5%_ (°C)	446.86	444.36	432.95	439.59
T_10%_ (°C)	455.32	452.82	448.41	451.71

**Table 7:** EEC values for virgin and aged samples.

Samples	R_p_ (MΩ.cm^−2^)	C_c_ (pF.cm^−2^)	Z_w_ (μΩ.cm^−2^)
Ref	95.5	17.4	
Exp 1	16.7	32.9	
Exp 2	3.83	78.7	1.62
Exp 3	12.8	23.6	1.68

**Table 8:** Dielectric loss of virgin and aged sample at lower frequencies.

Parameters	Ref ($\varepsilon^{\prime \prime }$)	Exp 1 ($\varepsilon^{\prime \prime }$)	Exp 2 ($\varepsilon^{\prime \prime }$)	Exp 3 ($\varepsilon^{\prime \prime }$)
Frequency (Hz)				
34.3043	0.0025	0.0075	5.67	0.157
6.73416	2.27 × 10^−18^	3.23 × 10^−17^	28.12	8.72 × 10^−16^
1.32193	2.80 × 10^−18^	8.34 × 10^−17^	142.25	4.21 × 10^−15^
0.00999989	0.567	12.845	12811	1.215× 10^−5^

## Data Availability

Data sharing is not applicable to this article.

## References

[1] Samimi A. (2013). Causes of Increased Corrosion in Oil and Gas Pipelines in the Middle East. Int. J. Basic Appl. Sci..

[2] Popoola L.T., Grema A.S., Latinwo G.K., Gutti B., Balogun A.S. (2013). Corrosion problems during oil and gas production and its mitigation. Int. J. Ind. Chem..

[3] Ghali E., Sastri V.S., Elboujdaini M. (2007). Corrosion Prevention and Protection: Practical Solutions.

[4] Papavinasam S. (2013). Corrosion Control in the Oil and Gas Industry.

[5] Groysman A. (2017). Corrosion Problems and Solutions in Oil Refining and Petrochemical Industry.

[6] Savino V., Mehdi M., Al-Dossary A. Thermoplastic Liners for Rehabilitation of Oil Flowline and Water Injection Lines, Integrity and Service Life. www.ndt.net/article/mendt2009/papers/Vincenzo-1.

[7] Esaklul K.A., Mason J. (2017). Nonmetallics applications in oil and gas production (pipes, liners, rehabilitations). Trends in Oil and Gas Corrosion Research and Technologies.

[8] Davis R., Snider B. (2017). Economically Mitigating Downhole Corrosion and Wear Failures with Thermoplastic Liners.

[9] Schweitzer P.A. (2000). Mechanical and Corrosion-Resistant Properties of Plastics and Elastomers.

[10] Ritums J.E., Mattozzi A., Gedde U.W., Hedenqvist M.S., Bergman G., Palmlöf M. (2006). Mechanical properties of high-density polyethylene and crosslinked high-density polyethylene in crude oil and its components. J. Polym. Sci. Part B Polym. Phys..

[11] Szklarz K.E., Baron J.J. (2004). Learnings from Thermoplastic Liner Failures in Sour Gas Pipeline Service and Replacement Liner Design and Installation.

[12] Zakaria N., Merican Z., Hamza M. (2019). Performance and Critical Issues of Polymer Liners in Pipeline Industry: A review. Mater. Today Proc..

[13] Brogden S., Lu L., Dowe A., Messina N., Robinson J. (2012). The use of Engineering Polymers for Internal Corrosion Protection of Hydrocarbon Pipelines. Proceedings of the MERL Oilfield Engineering with Polymers.

[14] Mierzwa-Hersztek M., Gondek K., Kopeć M. (2019). Degradation of polyethylene and biocomponent-derived polymer materials: An overview. J. Polym. Environ..

[15] Celina M.C. (2013). Review of polymer oxidation and its relationship with materials performance and lifetime prediction. Polym. Degrad. Stab..

[16] Hasegawa Y., Kusakabe K., Morooka S. (2001). Effect of temperature on the gas permeation properties of NaY-type zeolite formed on the inner surface of a porous support tube. Chem. Eng. Sci..

[17] Perez E.V., Balkus Jr K.J., Ferraris J.P., Musselman I.H. (2013). Instrument for gas permeation measurements at high pressure and high temperature. Rev. Sci. Instrum..

[18] Khalid H.U., Ismail M.C., Nosbi N. (2020). Permeation Damage of Polymer Liner in Oil and Gas Pipelines. Rev. Polym..

[19] Torres A.H.U., d’Almeida J.R.M., Habas J.-P. (2011). Aging of HDPE pipes exposed to diesel lubricant. Polym.-Plast. Technol. Eng..

[20] Grabmayer K., Wallner G.M., Beißmann S., Braun U., Steffen R., Nitsche D., Röder B., Buchberger W., Lang R.W. (2014). Accelerated aging of polyethylene materials at high oxygen pressure characterized by photoluminescence spectroscopy and established aging characterization methods. Polym. Degrad. Stab..

[21] Grabmann M.K., Wallner G.M., Grabmayer K., Nitsche D., Lang R.W. (2018). Aging behavior and lifetime assessment of polyolefin liner materials for seasonal heat storage using micro-specimen. Sol. Energy.

[22] Chen G., Yang Y., Zhou C., Zhou Z., Yan D. (2019). Thermal-oxidative aging performance and life prediction of polyethylene pipe under cyclic and constant internal pressure. J. Appl. Polym. Sci..

[23] Flaconnèche B., Martin J., Klopffer M.H. (2001). Permeability, diffusion and solubility of gases in polyethylene, polyamide 11 and poly (vinylidene fluoride). Oil Gas Sci. Technol..

[24] Menon N.C., Kruizenga A.M., Alvine K.J., San Marchi C., Nissen A., Brooks K. (2016). Behaviour of Polymers in High Pressure Environments as Applicable to the Hydrogen Infrastructure.

[25] Mason J.F., Stanley M., Ponda A., Demicoli D. (2017). Case Study: Engineered Polyamide 12 (PA12) Pipeline Liner for Management of Sour Gas Corrosion at Elevated Temperatures.

[26] Zhou J., Chen X. (2019). Compatibility study of high-density polyethylene with ethanol–gasoline and biodiesel. J. Elastomers Plast..

[27] Durbin T.D., Karavalakis G., Norbeck J.M., Park C.S., Castillo J., Rheem Y., Bumiller K., Yang J., Van V., Hunter K. (2016). Material compatibility evaluation for elastomers, plastics, and metals exposed to ethanol and butanol blends. Fuel.

[28] El-Sherik A. (2017). Trends in Oil and Gas Corrosion Research and Technologies: Production and Transmission.

[29] Technology F. Flexible Pipe Polymer Monitoring with Coupons. http://www.kks.com.au/wp-content/uploads/2014/01/Force-Technology-Pipe-Monitoring-flexible-pipe-polymer-monitoring-with-coupons.pdf.

[30] Kranbuehl D.E. (2009). Method to Predict the End-Point, Replacement Time and to Monitor Changes in that Time Using pre Aged Witness Coupons. Google Patents.

[31] Wolodko J., Fotty B., Perras T. (2016). Application of Non-Metallic Materials in Oil Sands Operations.

[32] Muren J., Caveny K., Eriksen M., Viko N.G., MÜLler-Allers J., JØRgen K. (2013). Un-Bonded Flexible Risers-Recent Field Experience and Actions for Increased Robustness.

[33] Lu F., Song B., He P., Wang Z., Wang J. (2017). Electrochemical impedance spectroscopy (EIS) study on the degradation of acrylic polyurethane coatings. RSC Adv..

[34] Tang Y., Cao J., Qu S., Quan L., Zhao X., Zuo Y. (2018). Degradation of a High Build Epoxy Primer/Polyurethane Composite Coatings under Cyclic Wet–dry Conditions. Int. J. Electrochem. Sci..

[35] Garcia E., Torres J., Rebolledo N., Arrabal R., Sanchez J. (2021). Corrosion of Steel Rebars in Anoxic Environments. Part I: Electrochemical Measurements. Materials.

[36] Yang Y., Akid R. (2015). Electrochemical investigation of the corrosion of different microstructural phases of X65 pipeline steel under saturated carbon dioxide conditions. Materials.

[37] Bozzini B., Gianoncelli A., Kourousias G., Boniardi M., Casaroli A., Dal Zilio S., Hussain R., Abyaneh M.K., Kiskinova M., Mele C. (2020). The role of chromium in the corrosion performance of cobalt-and cobalt-nickel based hardmetal binders: A study centred on X-ray absorption microspectroscopy. Int. J. Refract. Met. Hard Mater..

[38] Mele C., Bilotta A., Bocchetta P., Bozzini B. (2017). Characterization of the particulate anode of a laboratory flow Zn–air fuel cell. J. Appl. Electrochem..

[39] Jung M.R., Horgen F.D., Orski S.V., Rodriguez V., Beers K.L., Balazs G.H., Jones T.T., Work T.M., Brignac K.C., Royer S.-J. (2018). Validation of ATR FT-IR to identify polymers of plastic marine debris, including those ingested by marine organisms. Mar. Pollut. Bull..

[40] Prasad S.G., Lal C., Sahu K.R., Saha A., De U. (2021). Spectroscopic Investigation of Degradation Reaction Mechanism in γ-Rays Irradiation of HDPE. Biointerface Res. Appl. Chem..

[41] Fakirov S., Krasteva B. (2000). On the glass transition temperature of polyethylene as revealed by microhardness measurements. J. Macromol. Sci. Part B.

[42] Whelton A.J., Dietrich A.M. (2009). Critical considerations for the accelerated ageing of high-density polyethylene potable water materials. Polym. Degrad. Stab..

[43] Blaine R.L. (2002). Thermal Applications Note.

[44] Weon J.-I. (2010). Effects of thermal ageing on mechanical and thermal behaviors of linear low density polyethylene pipe. Polym. Degrad. Stab..

[45] Hamzah M., Khenfouch M., Rjeb A., Sayouri S., Houssaini D.S., Darhouri M., Srinivasu V.V. (2018). Surface Chemistry Changes and Microstructure Evaluation of Low Density Nanocluster Polyethylene under Natural Weathering: A Spectroscopic Investigation.

[46] Charles J. (2009). Qualitative analysis of high density polyethylene using FTIR spectroscopy. Asian J. Chem..

[47] Kong L., Fan X., Ding N., Ding H., Shao X., Li H., Qi D., Liu Q., Xu Y., Ge P. (2020). Experimental and Simulation Investigation on Failure Mechanism of a Polyethylene Elbow Liner Used in an Oilfield Environment. J. Fail. Anal. Prev..

[48] Nandiyanto A.B.D., Oktiani R., Ragadhita R. (2019). How to read and interpret FTIR spectroscope of organic material. Indones. J. Sci. Technol..

[49] Singh R.K., Ruj B., Sadhukhan A.K., Gupta P. (2020). A TG-FTIR investigation on the co-pyrolysis of the waste HDPE, PP, PS and PET under high heating conditions. J. Energy Inst..

[50] Brandon J., Goldstein M., Ohman M.D. (2016). Long-term aging and degradation of microplastic particles: Comparing in situ oceanic and experimental weathering patterns. Mar. Pollut. Bull..

[51] Guo S.-M., Yang Z.-G., Tang X.-Y., Zuo Y.-T. (2017). Safety assessment of high density polyethylene pipe with thermal damages. Plast. Rubber Compos..

[52] Bellamy L. (2013). The Infra-Red Spectra of Complex Molecules.

[53] Pagès P. (2005). Characterization of Polymer Materials Using FT-IR and DSC Techniques.

[54] Shirkavand M.J., Azizi H., Ghasemi I., Karabi M. (2018). Effect of Molecular Structure Parameters on Crystallinity and Environmental Stress Cracking Resistance of High-Density Polyethylene/TiO2 Nanocomposites. Adv. Polym. Technol..

[55] Mendes L.C., Rufino E.S., De Paula F.O.C., Torres A.C. (2003). Mechanical, thermal and microstructure evaluation of HDPE after weathering in Rio de Janeiro City. Polym. Degrad. Stab..

[56] Davidson R.G. (1992). Polymer degradation studies by FTIR. Progress in Pacific Polymer Science 2.

[57] Hadjiivanov K. (2014). Identification and characterization of surface hydroxyl groups by infrared spectroscopy. Adv. Catal..

[58] Khanam P.N., AlMaadeed M.A.A. (2015). Processing and characterization of polyethylene-based composites. Adv. Manuf. Polym. Compos. Sci..

[59] Cuadri A.A., Martín-Alfonso J.E. (2017). The effect of thermal and thermo-oxidative degradation conditions on rheological, chemical and thermal properties of HDPE. Polym. Degrad. Stab..

[60] Tantipattarakul S., Vaughan A.S., Andritsch T. (2020). Ageing behaviour of a polyethylene blend: Influence of chemical defects and morphology on charge transport. High Volt..

[61] Méndez-Hernández M.L., Tena-Salcido C.S., Sandoval-Arellano Z., González-Cantú M.C., Mondragón M., Rodríguez-González F.J. (2011). The effect of thermoplastic starch on the properties of HDPE/TPS blends during UV-accelerated aging. Polym. Bull..

[62] Shrivastava A. (2018). Introduction to Plastics Engineering.

[63] Teymouri Y., Adams A., Blümich B. (2018). Impact of Exposure Conditions on the Morphology of Polyethylene by Compact NMR.

[64] Khajehpour-Tadavani S., Nejabat G.-R., Mortazavi M.-M. (2019). Changes in crystallinity of HDPE films containing different amounts of an oxo-biodegradable additive due to UVC exposure. Polyolefins J..

[65] Ansaloni L., Alcock B., Peters T.A. (2020). Effects of CO_2_ on polymeric materials in the CO_2_ transport chain: A review. Int. J. Greenh. Gas Control..

[66] McKeen L.W. (2016). Permeability Properties of Plastics and Elastomers.

[67] Li D., Zhou L., Wang X., He L., Yang X. (2019). Effect of crystallinity of polyethylene with different densities on breakdown strength and conductance property. Materials.

[68] Yang R., Liu Y., Yu J., Wang K. (2006). Thermal oxidation products and kinetics of polyethylene composites. Polym. Degrad. Stab..

[69] Fu A., Zhao B., Yuan J., Yin C. (2019). Lab Research and Filed Experience of HDPE-Lined Tubing Used in Nitrogen Injection Well.

[70] Bredács M., Frank A., Bastero A., Stolarz A., Pinter G. (2018). Accelerated aging of polyethylene pipe grades in aqueous chlorine dioxide at constant concentration. Polym. Degrad. Stab..

[71] University K. Using Electrochemical Impedance Spectroscopy (EIS) for Evaluating Coating Performance in the Laboratory. https://kta.com/kta-university/using-electrochemical-impedance/.

[72] GAMRY Instruments EIS of Organic Coatings and Paints. https://www.gamry.com/application-notes/EIS/eis-of-organic-coatings-and-paints/.

[73] Joshi J.H., Kanchan D.K., Joshi M.J., Jethva H.O., Parikh K.D. (2017). Dielectric relaxation, complex impedance and modulus spectroscopic studies of mix phase rod like cobalt sulfide nanoparticles. Mater. Res. Bull..

[74] Marín-Genescà M., García-Amorós J., Mujal-Rosas R., Massagués L., Colom X. (2020). Study and Characterization of the Dielectric Behavior of Low Linear Density Polyethylene Composites Mixed with Ground Tire Rubber Particles. Polymers.

[75] Aziz S.B., Marf A.S., Dannoun E., Brza M.A., Abdullah R.M. (2020). The Study of the Degree of Crystallinity, Electrical Equivalent Circuit, and Dielectric Properties of Polyvinyl Alcohol (PVA)-Based Biopolymer Electrolytes. Polymers.

[76] Misra M., Kumar S.K. (2017). Using time–temperature superposition for determining dielectric loss in functionalized polyethylenes. ACS Macro Lett..

[77] Mikhaĭlov G.P., Sazhin B.I. (1960). Effect of Crystallization of Polymers on Dielectric Loss. Rubber Chem. Technol..

